# A preparation of murine liver fragments for *in vitro* studies: liver preparation for toxicological studies

**DOI:** 10.1186/1756-0500-6-70

**Published:** 2013-02-25

**Authors:** Ali S Alfazari, Bayan Al-Dabbagh, Saeeda Almarzooqi, Alia Albawardi, Abdul-Kader Souid

**Affiliations:** 1 Departments of Medicine, United Arab Emirates University, P.O. Box 15551, Al Ain, UAE; 2 Departments of Pathology, United Arab Emirates University, P.O. Box 15551, Al Ain, UAE; 3 Departments of Pediatrics, United Arab Emirates University, P.O. Box 15551, Al Ain, UAE

**Keywords:** *In vitro*, Cytotoxicity, Apoptosis, Liver, Mice, Cellular respiration, Bioenergetics, Caspases

## Abstract

**Background:**

The aim of this study was to develop liver tissue preparation suitable for investigating toxins. Hepatocyte respiration, ATP content, urea synthesis, caspase activity and morphology were measured as a function of *in vitro* incubation time. Mice were anesthetized by sevoflurane inhalation. Small liver fragments were then rapidly excised and incubated at 37°C in Krebs-Henseleit buffer (continuously gassed with 95% O_2_: 5% CO_2_) for up to 6 h. Phosphorescence O_2_ analyzer was used to determine the rate of cellular mitochondrial O_2_ consumption (*k*_*c*_, μM O_2_ min^-1^ mg^-1^). Cellular ATP was measured using the luciferin/luciferase system. The caspase-3 substrate *N*-acetyl-asp-glu-val-asp-7-amino-4-methylcoumarin (Ac-DEVD-AMC) was used to monitor intracellular caspase activity; cleaved AMC moieties (reflecting caspase activity) were separated on HPLC and detected by fluorescence.

**Findings:**

Respiration was inhibited by cyanide, confirming the oxidation occurred in the respiratory chain. The values of *k*_*c*_ (mean ± SD) for 0≤ *t* ≤6 h were 0.15 ± 0.02 μM O_2_ min^-1^ mg^-1^ (n = 18, coefficient of variation, CV = 13%), ATP content 131 ± 69 pmol mg^-1^ (1≤ *t* ≤6 h, n = 16, CV = 53%), synthesized urea 0.134 ± 0.017 mg/dL mg^-1^ in 50 min (0≤ *t* ≤6 h, n = 14, CV = 13%), and AMC peak area 62,540 ± 26,227 arbitrary units mg^-1^ (1≤ *t* ≤6 h, n = 3, CV = 42%). Hepatocyte morphology and organelles were reasonably persevered.

**Conclusions:**

The described liver tissue preparation demonstrates stable hepatocyte structure, ultrastructure and biomarkers for up to 6 h, permitting *in vitro* studies.

## Findings

Utilization of murine liver tissue for *in vitro* toxicological studies is recently reported [1-3]. In these studies, the measurements were limited to cellular mitochondrial O_2_ consumption (cellular respiration) and morphology. A more comprehensive assessment of hepatocyte functions, however, is frequently required. The present study includes additional hepatocyte biomarkers (ATP content, urea synthesis and caspase activity) in a preparation that is stable *in vitro* for up to 6 h.

The term cellular bioenergetics refers to the biochemical processes involved in energy metabolism (energy conversion or transformation). Cellular respiration, on the other hand, implies the delivery of O_2_ and metabolic fuels to the mitochondria, the oxidation of reduced metabolic fuels with passage of electrons to O_2_, and the synthesis of ATP. Impaired bioenergetics or respiration, thus, entails an interference with any of these processes.

Impairments in the cellular membranes, mitochondria or metabolic enzymes are expected to disrupt energy production within the cells. Cellular bioenergetics, thus, can be used as a biomarker of drug-induced cytotoxicities [4].

The term apoptosis describes cellular mechanisms responsible for initiating and executing cell death. The “initiation” process requires leakage of cytochrome c and other pro-apoptotic molecules from the mitochondrial intermembrane space into the cytosol. In the cytosol, cytochrome c binds to the apoptotic protease activating factor-1 (Apaf-1), triggering the cascade of caspases (a series of cysteine, aspartate-specific proteases) [5]. Caspase activation executes mitochondrial dysfunction, cellular membrane disruption (e.g., phosphatidylserine exposure) and DNA fragmentation. The mitochondrial perturbation involves opening the permeability transition pores (accelerating oxidations in mitochondrial respiratory chain) and collapsing the electrochemical potential [6]. Cytochrome c- and apoptosome-independent pathways for caspase activation are also known [7]. Thus, initiation and execution of apoptosis are directly linked to mitochondria. The fate of the cell is determined by the severity of cellular derangements and the capacity of cellular repair mechanisms. Cellular bioenergetics (ATP turnover) plays vital roles in repairing the caspase-induced damages [8]. Thus, the appearance of intracellular biomarkers for apoptosis does not necessarily indicate cell death.

Cells with intact bioenergetics are more capable of repairing toxic damages. Cellular ATP depletion, on the other hand, inevitably leads to cell death. Thus, apoptosis (intracellular caspase activity) is more likely to result in cell death if the cellular bioenergetics is impaired. This fact stems from the cellular dependency on aerobic metabolism. In contrast, cancer cells are more capable of surviving on anaerobic metabolism (a process termed “aerobic glycolysis” or Warburg effect) [9].

The study here assessed liver tissue bioenergetics (respiration and ATP content), urea synthesis, intracellular caspases, structure and ultrastructure in a preparation that is reasonably stable *in vitro* for up to 6 h. The measured biomarkers are suitable for *in vitro* investigation of effects of pathogens and toxins on the liver.

## Materials and methods

Pd(II) complex of *meso*-tetra-(4-sulfonatophenyl)-tetrabenzoporphyrin (Pd phosphor) was purchased from Porphyrin Products (Logan, UT). A lyophilized powder of caspase inhibitor I [*N*-benzyloxycarbonyl-val-ala-asp(O-methyl)-fluoromethylketone; zVAD-fmk; *m.w.* = 467.5; pan-caspase inhibitor] was purchased from Calbiochem (La Jolla, CA). Ac-DEVD-AMC (*N*-acetyl-asp-glu-val-asp-7-amino-4-methylcoumarin; *m.w.* = 675.64; caspase-3 substrate) was purchased from Axxora LLC (San Diego, CA). Glucose (anhydrous) and remaining reagents were purchased from Sigma-Aldrich (St. Louis, MO).

zVAD-fmk solution (2.14 mM) was made by dissolving 1.0 mg in 1.0 mL dimethyl sulfoxide and stored at -20°C in small aliquots. Ac-DEVD-AMC solution (7.4 mM) was made by dissolving 5.0 mg in 1.0 mL dimethyl sulfoxide and stored at -20°C. Pd phosphor solution (2.5 mg/ml = 2 mM) was prepared in dH_2_O and stored at -20°C in small aliquots. Sodium cyanide (NaCN) solution (1.0 M) was prepared in dH_2_O; the *p*H was adjusted to ~7.0 with 12 N HCl and stored at -20°C. Glucose oxidase (10 mg/mL) was dissolved in dH_2_O and stored at -20°C. Krebs-Henseleit (KH) buffer (115 mM NaCl, 25 mM NaHCO_3_, 1.23 mM NaH_2_PO_4_, 1.2 mM Na_2_SO_4_, 5.9 mM KCL, 1.25 mM CaCl_2_, 1.18 mM MgCl_2_ and 10 mM glucose, *p*H ~7.4) was made fresh.

### Animals

Male mice (Taylor outbred and C57Bl/6 strains, 8-10 weeks old, weighing about 25-30 grams) were used in this study. The animals were maintained at the animal facility that was in compliance with the National Institutes of Health guidelines (http://grants.nih.gov/grants/olaw/references/phspol.htm). All animals were housed in rooms maintained at 22°C with ~60% relative humidity and a 12-h light/dark cycle. They had *ad libitum* access to standard rodent chow and filtered water.

### Ethic statement

The study was approved by the Animal Ethics Committee - United Arab Emirates University - College of Medicine and Health Sciences. The study was carried out in strict accordance with the recommendations in the Guide for the Care and Use of Laboratory Animals of the National Institutes of Health. All surgery was performed under anesthesia, and all efforts were made to minimize suffering.

### Liver tissue

The animals were anesthetized by sevoflurane inhalation (100 μL per 10 g) and sacrificed as outlined [1,2]. Liver specimens (~20 to 30 mg) were collected using a 4-mm human skin biopsy punch (Miltex GmbH, Germany). Collected fragments were *immediately* immersed in continuously gassed with 95% O_2_: 5% CO_2_ ice-cold KH buffer. For O_2_ measurements, specimens were placed in 1.0 mL KH buffer containing 0.5% fat-free bovine albumin and 3 μM Pd phosphor. Specimens were also processed for measuring caspase activity and ATP content as described below.

### Intracellular caspase activity

Liver specimens were collected from mice as described [1]. The samples were incubated *in vitro* at 37°C in 50 mL KH buffer (continuously gassed with 95% O_2_: 5% CO_2_) for up to 6 h. At specific time points, samples were incubated in oxygenated KH buffer with 32 μM zVAD-fmk or 15 μL DMSO for 20 min (f/v = 1.0 mL). Ac-DEVD-AMC (37 μM) was then added and the incubation continued for additional 20 min. At the end of incubation, the tissue was disrupted by vigorous homogenization for 2 min, sonication for 3 min and 10 passages through a 27-G needle. The tissue disruption procedure quenched the Ac-DEVD-AMC cleavage reaction, mainly since caspases became practically inactive due to dilution. The supernatant was collected by centrifugation (~16,300 *g* for 90 min) through Microcentrifuge Filter (nominal molecular weight limit = 10,000 Dalton, Sigma^©^), separated on HPLC, and analyzed for the free fluorogenic AMC moiety.

### HPLC

The analysis was performed on a Waters reversed-phase HPLC system, which consisted of a manual injector, a pump and a fluorescent detector. The column, 4.6 × 250 mm Beckman Ultrasphere IP column, was operated at 25°C at 1.0 ml/min. For AMC detection, the excitation wavelength was 380 nm and the emission wavelength 460 nm. Solvents A and B were HPLC-grade methanol:dH_2_O 1:1 (isocratic). The run time was 20 min and the injection volume was 20 μL.

### Oxygen measurement

A phosphorescence O_2_ analyzer was used to monitor O_2_ consumption by liver specimens [1,2]. Briefly, O_2_ detection was performed with the aid of Pd phosphor (absorption maximum at 625 nm and phosphorescence maximum at 800 nm). Samples were exposed to light flashes (600 per min) from a pulsed light-emitting diode array with peak output at 625 nm (OTL630A-5-10-66-E, Opto Technology, Inc., Wheeling, IL). Emitted phosphorescent light was detected by a Hamamatsu photomultiplier tube after passing through an interference filter centered at 800 nm. The amplified phosphorescence decay was digitized at 1.0 MHz by a 20-MHz A/D converter (Computer Boards, Inc., Mansfield, MA).

A program was developed using Microsoft Visual Basic 6, Microsoft Access Database 2007, and Universal Library components (Universal Library for Measurements Computing Devices; http://www.mccdaq.com/daq-software/universal-library.aspx). It allowed direct reading from the PCI-DAS 4020/12 I/O Board (PCI-DAS 4020/12 I/O Board; http://www.mccdaq.com/pci-data-acquisition/PCI-DAS4020-12.aspx). The pulse detection was accomplished by searching for 10 phosphorescence intensities >1.0 volt (by default). Peak detection was accomplished by searching for the highest 10 data points of a pulse and choosing the data point closest to the pulse decay curve [10].

The phosphorescence decay rate (1/τ) was characterized by a single exponential; I = Ae^-*t*/τ^, where I = Pd phosphor phosphorescence intensity. The values of 1/τ were linear with dissolved O_2_: 1/τ = 1/τ^o^ + *k*_*q*_[O_2_], where 1/τ = the phosphorescence decay rate in the presence of O_2_, 1/τ^o^ = the phosphorescence decay rate in the absence of O_2_, and *k*_q_ = the second-order O_2_ quenching rate constant in s^-1^ • μM^-1^[11].

Liver tissue respiration was measured at 37°C in 1-mL sealed vials. Mixing was with the aid of parylene-coated stirring bars. In vials sealed from air, [O_2_] decreased linearly with time, indicating the kinetics of mitochondrial O_2_ consumption was zero-order. The rate of respiration (*k*, in μM O_2_ min^-1^) was thus the negative of the slope d[O_2_]/d*t*. NaCN inhibited respiration, confirming O_2_ was being consumed in the mitochondrial respiratory chain.

The calibration reaction contained PBS with 3 μM Pd phosphor, 0.5% fat-free albumin, 50 μg/mL glucose oxidase and various concentrations of β-glucose. [O_2_] was calculated using, 1/τ = 1/τ^o^ + *k*_*q*_[O_2_] [11].

### Cellular ATP content

Liver tissue fragments were homogenized in 0.5 mL of ice-cold 2% trichloroacetic acid for 2 min. The supernatants were collected by centrifugation (1000 × *g* at 4°C for 5 min) and stored at -20°C until analysis. Immediately before ATP measurement, the samples were thawed and neutralized with 0.5 mL 100 mM Tris-acetate, 2 mM EDTA (final *p*H, 7.75). ATP concentration was determined using the Enliten ATP Assay System (Bioluminescence Detection Kit, Promega, Madison, WI). Briefly, 2.5 μL of the supernatant was added to 25 μL of the luciferin/luciferase reagent. The luminescence intensity was then measured at 25°C using Glomax Luminometer (Promega, Madison, WI). The ATP standard curve ranged from 10 pM to 100 nM (*R*^*2*^ >0.9999).

### Urea synthesis

Liver specimens were incubated at 37°C in 50 ml KH buffer continuously gassed with 95% O_2_: 5% CO_2_. At indicated time points, specimens (~80 mg each) were taken from the incubation solution and placed in 1.0 mL KH buffer supplemented with 10 mM NH_4_Cl and 2.5 mM ornithine. The reactions were allowed to continue at 37°C for 50 min with continuous gassing as above. At the end of the incubation period, the specimens were discarded and the solution was analyzed for urea as described [12]. Blood urea nitrogen (BUN, mmol/L) was measured using standard laboratory methods with an LX20 multiple automated analyzer (Beckman Coulter, CA, USA). For conversion, BUN (mg/dL) = BUN (mmol/L) ÷ 0.357; Urea (mg/dL) = BUN (mg/dL) × 2.14.

A second protocol was employed, which involved incubating liver specimens (small fragments weighing about 300 mg) at 37°C in 50 ml KH buffer supplemented with 10 mM NH_4_Cl and 2.5 mM ornithine and continuously gassed as above. At 0, 3 and 6 hr, an aliquot of the solution was taken for urea determination.

### Light microscopy

Liver samples were fixed in 10% neutral formalin, dehydrated in increasing concentrations of ethanol, cleared with xylene and embedded in paraffin. Three micrometer sections were prepared from paraffin blocks and stained with haematoxylin and eosin.

### Electron microscopy

Liver samples were immersed in McDowell and Trump fixative for 3 h at room temperature. Tissues were rinsed with phosphate buffer saline and fixed with 1% osmium tetroxide for 1 h. Samples were washed with dH_2_O, dehydrated in a series of graded ethanol and propylene oxide, infiltrated, embedded in Agar-100 epoxy resin and polymerized at 65°C for 24 h. Blocks were trimmed and semi-thin and ultrathin sections were cut with Reichert Ultracuts, ultra-microtome. The semi-thin sections (1 mm) were stained with 1% aqueous toluidine blue on glass slides. The ultrathin sections (95 nm) on 200 mesh Cu grids were contrasted with uranyl acetate followed by lead citrate double stain. The grids were examined and photographed under a Philips CM10 transmission electron microscope.

*Liver tissue preparation from Taylor outbred mice (specimens were incubated in KH buffer with continuous gassing with 95% O*_*2*_*: 5% CO*_*2*_*for up to 6 h).*

**Figure 1:**
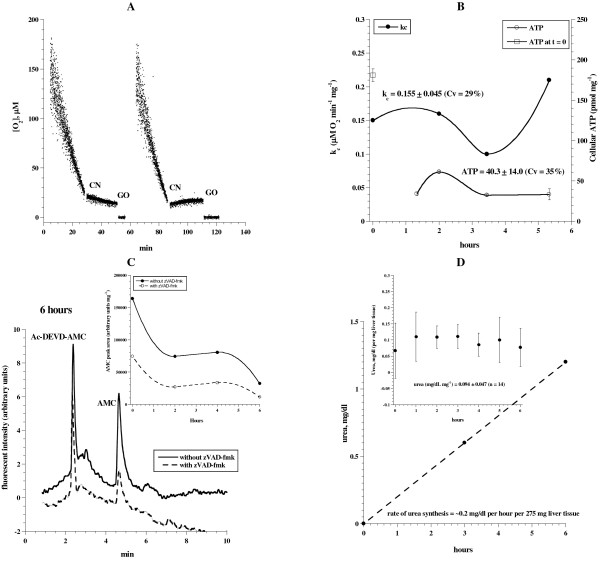
**Liver tissue respiration, ATP content, caspase activity and urea synthesis in continuously oxygenated KH buffer. **Liver specimens from a Taylor outbred mouse were incubated at 37°C in 50 mL KH buffer continuously gassed with 95% O_2_: 5% CO_2_ for up to ~6 h. At indicated time periods, samples were removed from the incubation medium and processed for measurements of O_2_ consumption, ATP content, caspase activity and urea synthesis as described in Methods. *Panel ***A**: Representative runs of cellular mitochondrial O_2_ consumption are shown; *t* = 0 corresponds to animal sacrifice. The rate of respiration (*k,* μM O_2_ min^-1^) was the negative of the slope of [O_2_] *vs. t*. The additions of 10 mM NaCN and 50 μg/ml glucose oxidase are shown. *Panel***B**: The values of *k*_*c*_ and ATP were plotted as a function of incubation time. *Panel ***C**: HPLC runs of caspase activity at 6 h. The retention time (*R*_t_) for Ac-DEVD-AMC was ~2.5 min and AMC (representing caspase activity) ~4.8 min. *Panel ***C***insert*: AMC peak areas are shown as a function of incubation time. *Panel***D**: Liver specimens (~300 mg) were incubated at 37°C in 50 ml KH buffer supplemented with 10 mM NH_4_Cl and 2.5 mM ornithine and continuously gassed with 95% O_2_: 5% CO_2_. At *t* = 0, 3 and 6 h, an aliquot of the solution was taken for urea determination. Insert (error bars are standard deviation of 2 independent experiments), liver specimens were incubated at 37°C in 50 ml KH buffer continuously gassed as above. At indicated time points, specimens (~80 mg) were placed in 1.0 mL KH buffer supplemented with 10 mM NH_4_Cl and 2.5 mM ornithine. The reactions were allowed to continue at 37°C for 50 min with continuous gassing as above. At the end of the incubation period, the specimens were discarded and the solution was analyzed for urea.

Liver fragments were collected as above and incubated at 37°C in 50 mL KH buffer continuously gassed with 95% O_2_: 5% CO_2_ for up to ~6 h. At indicated time periods (Figure 1), samples were removed from the incubation medium and processed for measurements of cellular mitochondrial O_2_ consumption (expressed as, *k*_*c*_ in μM O_2_ min^-1^ mg^-1^), ATP content (pmol mg^-1^), caspase activity (arbitrary units mg^-1^) and urea synthesis (mg/dL mg^-1^). Figure 1A shows representative runs of O_2_ consumption that were inhibited by cyanide (CN), confirming the oxidation occurred in the mitochondrial respiratory chain. The addition of glucose oxidase (GO) depleted the remaining O_2_ in the solution. The values (mean ± SD) of *k*_*c*_ for 0 ≤ *t* ≤ 6 h were 0.155 ± 0.045 (n = 4; coefficient of variation, CV = 29%), Figure 1B. The corresponding values of cellular ATP (Figure 1B, done in triplicates) for 1 ≤ *t* ≤ 6 h were 40.3 ± 14.0 pmol mg^-1^ (n = 4; CV = 35%; please note the value of ATP at *t* = 0 was 181.1 ± 8.0). These results show a reasonable *in vitro* stability of hepatocyte oxidative phosphorylation for about 6 h. The sharp declined in cellular ATP in the first hour demonstrated an inability to fully sustain the *in vivo* status of hepatocyte bioenergetics.

Representative HPLC run for caspase activity is shown in Figure 1C. Figure 1C (insert) shows a relatively low AMC peak area (reflecting caspase activity) at 6 h. AMC peak areas in the presence of the pancaspase inhibitor zVAD-fmk were reduced by 55 to 65% (Figure 1C), confirming the cleavage reaction was mainly due to caspase activity.

Urea synthesis by liver specimens is shown in Figure 1D. A steady increase in the rate of urea synthesis was demonstrated up to 6 h. Briefly, liver specimens (~300 mg) were incubated at 37°C in 50 ml KH buffer supplemented with 10 mM NH_4_Cl and 2.5 mM ornithine and continuously gassed with 95% O_2_: 5% CO_2_. At *t* = 0, 3 and 6 h, an aliquot of the solution was taken for urea determination. The rate of urea synthesis was ~0.2 mg/dL per h per 275 mg.

In Figure 1D (insert), liver specimens were incubated at 37°C in 50 ml KH buffer continuously gassed as above. At indicated time points, specimens (~80 mg) were placed in 1.0 mL KH buffer supplemented with 10 mM NH_4_Cl and 2.5 mM ornithine. The reactions were allowed to continue at 37°C for 50 min with continuous gassing as above. At the end of the incubation period, the specimens were discarded and the solution was analyzed for urea. The concentrations of urea (over 50 min; 2 independent experiments) were 0.134 ± 0.017 mg/dL mg^-1^ (0 <*t* ≤ 6 h, n = 14, CV = 13%). Thus, urea synthesis by the preparations was stable for up 6 h. Similar results were obtained for the C57 strain of mice.

**Figure 2:**
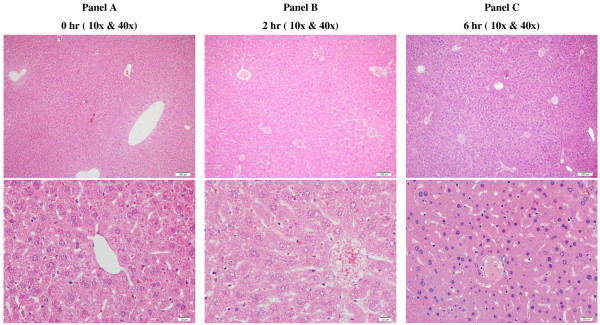
**Liver histology (oxygenated KH buffer). **Normal hepatic architecture characterized by polygonal hepatocytes arranged in plates that radiate from central vein towards portal tracts. Hepatic plates are separated by thin sinusoids. Comparisons of hepatic architecture and cellular morphology in **A** to **C** demonstrate good preservation of cellular membranes and nuclear chromatin details over a period of 6 h. (H&E, 10x & 40x).

Figure 2 shows normal polygonal hepatocytes arranged in plates that radiate from central vein towards portal tracts. The hepatic plates were separated by thin sinusoids. Comparisons of the architecture between *t* = 0, t = 2 h and *t* = 6 h demonstrate a good preservation of the hepatic structure over 6 h.

**Figure 3:**
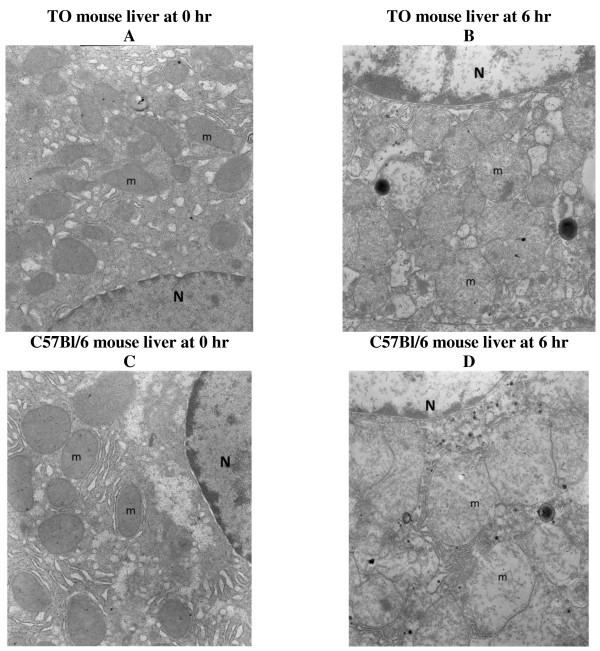
**Electron microscopy images (oxygenated KH buffer). **(**A**) TO mouse liver at 0 h demonstrating a hepatocyte with preserved architecture. Note hepatocyte nucleus (N) and numerous intact mitochondria (m). (**B**) TO mouse liver at 6 h demonstrating a preserved hepatocyte microarchitecture with only mild swelling of mitochondria (m). Note hepatocyte nucleus (N). (**C**) C57Bl/6 mouse liver at time 0 demonstrating a hepatocyte with preserved architecture. Note hepatocyte nucleus (N) and numerous intact mitochondria (m). (**D**) C57Bl/6 mouse liver at time 6 demonstrating a hepatocyte with swollen mitochondria (m). Note hepatocyte nucleus (N). Magnification 140,000.

The electron microscopy images also confirmed preserved hepatocyte microarchitecture, but with mild swelling of the mitochondria at 6 h in both TO and C57Bl/6 mouse strains (Figure 3).

*Liver tissue preparation from* C57Bl/6 *mice (specimens were incubated in KH buffer without continuous oxygenation for up to 6 h).*

**Figure 4:**
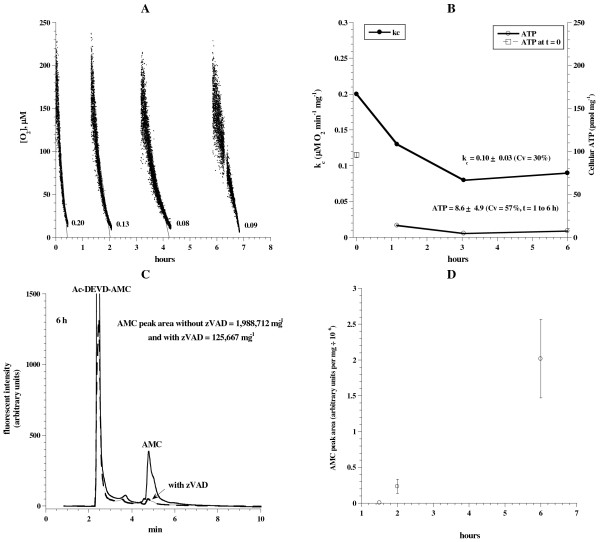
**Liver tissue respiration, ATP content and caspase activity in unoxygenated KH buffer. **Liver specimens from a C57Bl/6 mouse were incubated at 37°C in 50 mL KH buffer for up to ~6 h. At indicated time periods, samples were removed from the incubation medium and processed for measurements of O_2_ consumption, ATP content and caspase activity as described in Methods. *Panel ***A**: Representative runs of cellular mitochondrial O_2_ consumption are shown; *t* = 0 corresponds to animal sacrifice. The rate of respiration (*k,* μM O_2_ min^-1^) was the negative of the slope of [O_2_] *vs. t*. *Panel***B**: The values of *k*_*c*_ and ATP are plotted as a function of incubation time. *Panel ***C**: HPLC runs of caspase activity at 6 h with and without the pancaspase inhibitor zVAD-fmk. *Panel ***D**: AMC peak areas (retention time, ~4.8 min) are shown as a function of incubation time.

**Figure 5:**
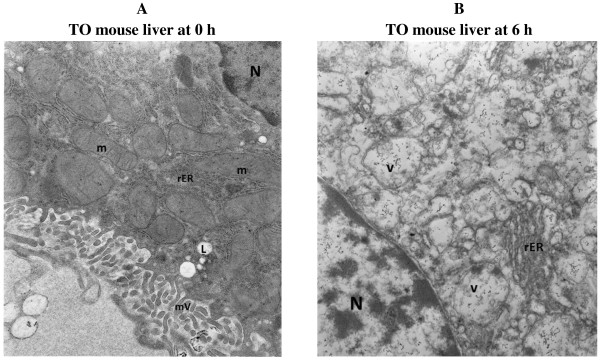
**Electron microscopy images (unoxygenated KH buffer). **(**A**) Liver at time 0 demonstrating hepatocytes with preserved architecture. Note hepatocyte nucleus (N), numerous intact mitochondria (m), rough endoplasmic reticulum (rER), microvilli (mV) and cytoplasmic lipid vacuoles (L). (**B**) Liver at 6 h demonstrating hepatocytes with numerous cytoplasmic vesicles (v), rough endoplasmic reticulum (rER) and a nucleus (N). Some of the vesicles probably represent swollen disintegrating mitochondria under experimental conditions. Magnification 140,000.

The need for continuous gassing with O_2_: CO_2_ was investigated in C57Bl/6 mice. Liver fragments were incubated at 37°C in 50 mL KH buffer *without* continuous gassing with 95% O_2_: 5% CO_2_. At indicated time periods (Figure 4), samples were removed from the incubation medium and processed for the measurements described for Figure 1. Figure 4A-B shows representative runs of O_2_ consumption. The rate of respiration progressively decreased with time (*k*_*c*_ = 0.2 μM O_2_ min^-1^ mg^-1^ at *t* =0 h and 0.09 at *t* = 6 h). This more than 50% reduction in the respiration was also accompanied by relatively low cellular ATP content. For 1 <*t* ≤ 6 h, cellular ATP was 8.8 ± 4.9 pmol mg^-1^ (Figure 4B), which was 21% of that shown with continuous oxygenation (Figure 1B). Consistently, intracellular caspase activity markedly increased at hour 6 (Figure 4C-D). The AMC peak area at 6 h without zVAD-fmk was 1,988,712 and with zVAD-fmk was 125,667 (Figure 4C). The corresponding values with continuous oxygenation were 32,477 and 11,372, respectively (see Figure 1C). Thus, caspase activity increased about 6-fold without oxygenation. These results showed the link between hepatocyte bioenergetics and induction of caspases; that is, increased caspase activity was associated with impaired cellular bioenergetics. EM images of the liver at *t* = 0 demonstrated hepatocytes with preserved architecture, nucleus, mitochondria, rough endoplasmic reticulum, microvilli and cytoplasmic lipid vacuoles (Figure 5A). Numerous cytoplasmic vesicles, however, were present at *t* = 6 h, representing swollen disintegrating mitochondria under the experimental conditions (Figure 5B).

### Significance

The main purpose of this work was to develop *in vitro* method suitable for studying the effects of toxins and drugs on hepatocyte metabolic biomarkers (cellular respiration, ATP content, caspase activity and urea synthesis). The preparation described in Figure 1 resulted in reasonable preservation of liver bioenergetics with minimum caspase activity for up to 6 h. The response of this *in vitro* system is demonstrated by induction of intracellular caspases by the hepatotoxin dactinomycin (Additional file 1: Figure S1, Supplementary Materials).

The success of this procedure relies on three important steps. The first step involves rapid (within 30 sec) excision of thin liver fragments while the organ is still perfused. The second step involves *immediate* immersion of the excised tissue in *ice-cold* KH buffer (continuously gassed with 95% O_2_: 5% CO_2_). If needed, specimens were cut into smaller slices using a 4-mm punch biopsy device while in the oxygenated ice-cold buffer. The third step involves incubating the fragments in a large volume (e.g., 50 mL) of KH buffer with continuous gassing with 95% O_2_: 5% CO_2_. Having adhered to these steps, the coefficient of variation for the rate of respiration was 13% (0 ≤ *t* ≤ 6 h), ATP content 53% (1 ≤ *t* ≤ 6 h), and urea synthesis 13% (0 ≤ *t* ≤ 6 h).

Cellular ATP content was significantly higher at *t* = 0 (Figure 1B, open square), reflecting the inability to maintain the *in vivo* level of hepatocyte ATP. Despite this limitation, the remaining parameters (hepatocyte respiration, urea synthesis, caspase activity, histology and organelles) were relatively preserved over 6 h (Figures 1, 2, 3).

As noted in Figures 4, 5, cellular ATP was depleted and cellular respiration was markedly decreased in unoxygenated buffer. These changes were accompanied by a “storm” of intracellular caspase activity, which is known to disturb mitochondrial functions (“the mitochondrial cell death pathway”).

The findings here highlight the sensitivity of hepatocytes to hypoxia and poor tissue perfusion. As shown (Figures 4, 5), the relative tissue hypoxia induced a collapse in hepatocyte bioenergetics (impaired respiration and ATP production) and caspase induction, which further impede cellular function and repair.

Active caspases permeabilize the inner mitochondrial membranes, resulting in a collapse in the proton motive force, loss of electrochemical potential and uncoupling of oxidative phosphorylation [5]. This process leads to rapid depletion of cellular nutrients, metabolic fuels and ATP. These consequences contribute to further hepatocyte damages. Thus, the initial cellular insults become compounded by subsequent mitochondrial derangements.

Another limitation of this preparation is the relative short (6 h) *in vitro* incubation. Longer experiments produced air bubbles in the hepatocytes and bacterial overgrowth. Future studies are needed to overcome these technical limitations.

In summary, the described method is relatively simple and requires minimal tissue handling. It permits comprehensive *in vitro* analyses for up to 6 h. Its main advantage is using one animal for multiple biomarkers. Other advantages include evading extensive tissue manipulation and collagenase digestion required for single cell preparations.

## Abbreviations

KH: Krebs-Henseleit; Ac-DEVD-AMC: the caspase-3 substrate *N*-acetyl-asp-glu-val-asp-7-amino-4-methylcoumarin; CV: Coefficient of variation; Pd phosphor: Pd(II) complex of *meso*-tetra-(4-sulfonatophenyl)-tetrabenzoporphyrin; zVAD-fmk: Caspase inhibitor I [*N*-benzyloxycarbonyl-val-ala-asp(O-methyl)-fluoromethylketone]; NaCN: Sodium cyanide; TO: Taylor outbred; 1/τ: Phosphorescence decay rate; BUN: Blood urea nitrogen; H&E: Haematoxylin and eosin;k: Rate of cellular respiration (in μM O_2_ min^-1^); kc: Corrected rate of cellular respiration (in μM O_2_ min^-1^ mg^-1^); GO: Glucose oxidase.

## Competing interests

The authors declare that they have no competing interests.

## Authors’ contributions

ASA and BA designed the study, carried out the analysis, interpreted the data and drafted the manuscript. SA and AA performed the histology. AKS supervised the progress and critically revised the manuscript. All authors read and approved the final manuscript.

## Supplementary Material

Additional file 1: Figure S1 HPLC runs of liver tissue caspase activity with and without 8 μM dactinomycin. Two independent experiments are shown. Liver fragments (40 mg each) from C57Bl/6 mice were incubated in 0.5 mL oxygenated KH buffer supplemented with 74 μM DEVD-AMC with (dashed lines) and without (solid lines) 42.8 μM zVAD-fmk. The incubations were allowed to continue at 37°C with and without 8 μM dactinomycin for 60 min. The tissues were then disrupted by vigorous homogenization for 2 min, sonication for 3 min and 10 passages through a 27-G needle. The supernatants were collected by centrifugation (~16,300 *g* for 90 min) through Microcentrifuge Filter (nominal molecular weight limit = 10,000 Dalton, Sigma^©^), separated on HPLC, and analyzed for the free fluorogenic AMC moiety. The analysis was performed on a Waters reversed-phase HPLC system. The column, 4.6 × 250 mm Beckman Ultrasphere IP column, was operated at 25°C at 1.0 ml/min. For AMC detection, the excitation wavelength was 380 nm and the emission wavelength 460 nm. The running solvents (isocratic) were HPLC-grade CH_3_CN:H_2_O [1:3, v/v] (Solvent A) and dH_2_O (Solvent B). The *R*_t_ for Ac-DEVD-AMC was ~4.5 min and AMC ~18.5 min. The AMC peak areas with and without zVAD are shown. Click here for file
