# Supplementary material for: A preparation of murine liver fragments for in vitro studies: liver preparation for toxicological studies
Source: BMC Res Notes. 2013 Feb 25;6:70. doi: 10.1186/1756-0500-6-70 (PMC3598918; doi:10.1186/1756-0500-6-70)
Supplement: Additional file 1: Figure S1 — HPLC runs of liver tissue caspase activity with and without 8 μM dactinomycin. Two independent experiments are shown. Liver fragments (40 mg each) from C57Bl/6 mice were incubated in 0.5 mL oxygenated KH buffer supplemented with 74 μM DEVD-AMC with (dashed lines) and without (solid lines) 42.8 μM zVAD-fmk. The incubations were allowed to continue at 37°C with and without 8 μM dactinomycin for 60 min. The tissues were then disrupted by vigorous homogenization for 2 min, sonication for 3 min and 10 passages through a 27-G needle. The supernatants were collected by centrifugation (~16,300 g for 90 min) through Microcentrifuge Filter (nominal molecular weight limit = 10,000 Dalton, Sigma©), separated on HPLC, and analyzed for the free fluorogenic AMC moiety. The analysis was performed on a Waters reversed-phase HPLC system. The column, 4.6 × 250 mm Beckman Ultrasphere IP column, was operated at 25°C at 1.0 ml/min. For AMC detection, the excitation wavelength was 380 nm and the emission wavelength 460 nm. The running solvents (isocratic) were HPLC-grade CH3CN:H2O [1:3, v/v] (Solvent A) and dH2O (Solvent B). The Rt for Ac-DEVD-AMC was ~4.5 min and AMC ~18.5 min. The AMC peak areas with and without zVAD are shown. [file 1756-0500-6-70-S1.doc]

| **A** | **B** |
| --- | --- |
| **C** | **D** |
